# Neuronal Interferon Signaling Is Required for Protection against Herpes Simplex Virus Replication and Pathogenesis

**DOI:** 10.1371/journal.ppat.1005028

**Published:** 2015-07-08

**Authors:** Pamela C. Rosato, David A. Leib

**Affiliations:** Department of Microbiology and Immunology, Geisel School of Medicine at Dartmouth, Lebanon, New Hampshire, United States of America; Northwestern University, UNITED STATES

## Abstract

Interferon (IFN) responses are critical for controlling herpes simplex virus 1 (HSV-1). The importance of neuronal IFN signaling in controlling acute and latent HSV-1 infection remains unclear. Compartmentalized neuron cultures revealed that mature sensory neurons respond to IFNβ at both the axon and cell body through distinct mechanisms, resulting in control of HSV-1. Mice specifically lacking neural IFN signaling succumbed rapidly to HSV-1 corneal infection, demonstrating that IFN responses of the immune system and non-neuronal tissues are insufficient to confer survival following virus challenge. Furthermore, neurovirulence was restored to an HSV strain lacking the IFN-modulating gene, γ34.5, despite its expected attenuation in peripheral tissues. These studies define a crucial role for neuronal IFN signaling for protection against HSV-1 pathogenesis and replication, and they provide a novel framework to enhance our understanding of the interface between host innate immunity and neurotropic pathogens.

## Author Summary

Herpes simplex virus type 1 (HSV-1) is a ubiquitous virus that can cause cold sores, blindness, and even death from encephalitis. There is no vaccine against HSV, and although antiviral drugs can control HSV-1, it persists because it establishes lifelong latent infections in neurons. Humans with deficiencies in innate immunity have significant problems controlling HSV infections. In this study we therefore sought to elucidate the role of neuronal innate immunity in the control of viral infection. Sensory neurons, in which HSV resides, have projection which that extend long distances to innervate the skin, the initial site of HSV infection. We found that neurons can respond to interferon beta, a molecule that strongly stimulates innate immunity and inhibits virus growth, at both the cell body and at the end of these long projections. Moreover, we found that this interferon response of neurons is critical for controlling HSV infection in vivo and that the interferon responses of non-neuronal cells are insufficient to provide protection. Our results have important implications for understanding how the nervous system defends itself against virus infections.

## Introduction

Herpes simplex virus type I (HSV-1) is a highly prevalent neurotropic virus that persists for the lifetime of the host. Upon initial infection, HSV-1 undergoes rounds of lytic replication in the peripheral orofacial mucosa. The virus then enters axon terminals of innervating sensory neurons and travels in a retrograde manner to the neuronal cell bodies of the trigeminal ganglia (TG). While the virus may subsequently undergo round-trip zosteriform spread from the infected TG back to the periphery [1], it is ultimately within sensory neurons that HSV-1 establishes latency, producing little to no infectious virus. Reactivation from latency can occur and HSV-1 travels in an anterograde direction down the axon of sensory neurons to the periphery where it undergoes subsequent rounds of lytic replication, enabling viral shedding and host-to-host spread [2].

The ability of HSV to establish latency enables persistence in the host, resulting in 65–90% seroprevalence [3]. In most cases, HSV-1 infection results in oral lesions or is largely asymptomatic. A minority of infected individuals, however, can develop herpes stromal keratitis (HSK), which can lead to blindness. In rare cases, herpes simplex encephalitis (HSE) can occur, which often results in death or long-term cognitive deficits. While HSE can result from primary infection, mostly in newborns, both disease pathologies can result from reactivation of latent HSV which then travels to the eye or CNS [4,5]. Nucleoside analogs such as acyclovir (ACV) reduce HSE mortality significantly, but survivors are often left with long-term neurological sequelae, and ACV cannot eliminate the latent virus reservoir [5].

The interferon (IFN)-driven antiviral response is critical for controlling HSV infection [6,7] and this response is initiated when infected cells detect the presence of virus through pattern recognition receptors (PRRs). PRRs signal through adaptor molecules, which go on to phosphorylate key transcription factors, namely IRF3 and IRF7, resulting in an up-regulation of type I interferon (IFN α and β). Type I IFN is then secreted from the cell and can signal IFN receptors on both infected and uninfected cells. This activates a JAK/STAT pathway through the transcription factor STAT1, leading to the establishment of an antiviral state through transcriptional repression, cytokine upregulation, and apoptosis [8]. Mice lacking components of antiviral signaling, such as IFN receptors or STAT1, have increased susceptibility to HSV infection [7,9]. This is mirrored in humans with genetic impairments in antiviral signaling who suffer increased frequency of recurrent HSE [6,10].

HSV counteracts the antiviral response through several proteins, underscoring the importance of antiviral signaling to both host and pathogen [11]. A key HSV protein that can counteract antiviral signaling is ICP34.5, encoded by the gene γ34.5. ICP34.5 prevents the phosphorylation of IRF3 and reverses the phosphorylation of eIF2α thereby relieving translational arrest [12–14]. ICP34.5 also inhibits autophagy, a process which can degrade intracellular virions, and potentiate antigen presentation [15,16]. Consistent with this, viruses lacking ICP34.5 are significantly attenuated in both humans [17] and animal models [18–20].

While IFN-driven antiviral signaling controls HSV infection in general, its specific role in neurons remains unclear. It is thought that neurons may lack robust innate immune signaling in order to avoid damage to a largely irreplaceable cell type [21]. Supporting this, work from Yordy and colleagues suggest that autophagy, not IFN signaling, is the dominant antiviral strategy employed by neurons to control HSV infection [21]. Consistent with this, we have shown that the intrinsic IFN-driven antiviral response of adult sensory neurons is impaired. We have also, however, demonstrated that paracrine IFN signaling can drive an effective antiviral response in neurons which is strongly countered by HSV ICP34.5 [22]. Consistent with these data there is mounting evidence for effective neuronal antiviral responses to several viruses [23–26]. Of relevance, IFN treatment of cultured neurons restricts HSV replication and promotes a quiescent state resembling latency [27,28]. Additionally, neurons derived from humans who suffer from recurrent HSE due to genetic defects in TLR3 signaling, are more permissive to HSV infection. These studies provide further evidence for a key role for neuronal antiviral signaling in controlling HSV [29]. A confounding aspect when interpreting these data, however, is that recent studies have highlighted the importance of differentiation state and neuronal subtype on antiviral signaling [23,30,31]. Taken together, this body of work led us to investigate the role of IFN signaling in mature sensory neurons during HSV-1 infection.

We therefore established a culture system of purified TG neurons from adult mice grown in compartmentalized chambers [32]. This model allowed us to mimic the *in vivo* axonal route of HSV-1 infection while enabling independent manipulation of the soma and axon of a relevant neuronal population. Using this *in vitro* system, we showed that the administration of IFNβ at either the soma or axon is capable of restricting HSV-1. To address the importance of neural IFN signaling *in vivo*, we employed a Cre-lox system to yield progeny mice with defects in STAT1-driven signaling specifically in neural tissue. Using these mice, we show that neural IFN signaling alone is necessary to control HSV-1 replication, disease and survival, and demonstrate restoration of virulence to a virus lacking IXΠ34.5. Correspondingly, non-neuronal IFN signaling is insufficient to control HSV-1 dissemination and mortality. Together, these results demonstrate that neuronal IFN signaling is required for controlling HSV-1 replication and disease and establish a new animal model for studying the role of neuronal innate immunity in the pathogenesis of neurotropic infections.

## Results

### Neuronal paracrine IFNβ signaling at the soma and distal axon controls HSV-1 upon axonal infection

**Fig 1:**
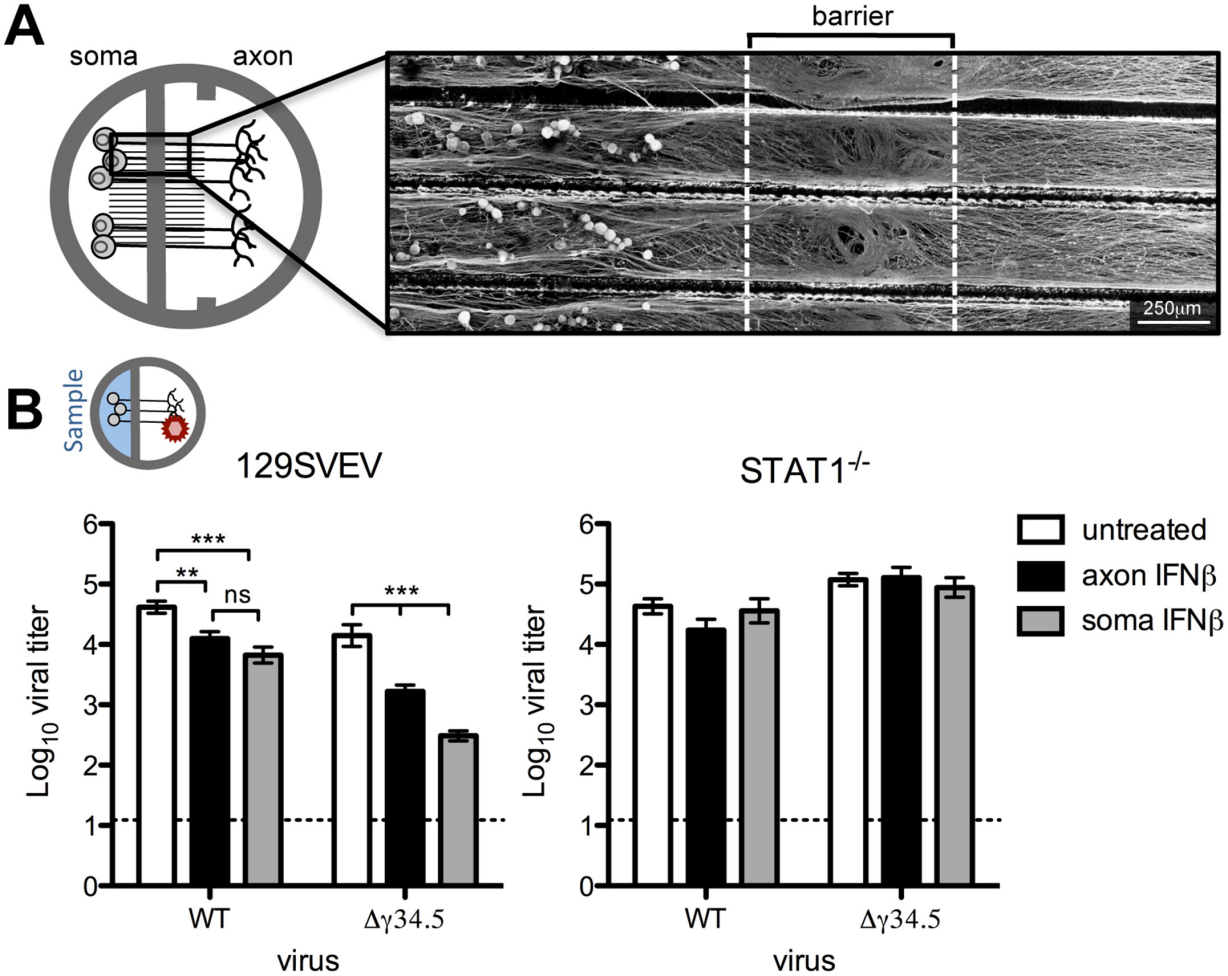
Paracrine IFNβ signaling at the cell body and distal axon reduces HSV-1 titers upon axonal infection. A) Diagram of Campenot chambers that were modified by removing one of two central barriers. Trigeminal ganglia (TG) neurons from adult mice were isolated and seeded in soma compartment. Micrograph shows βIII-tubulin staining of neurons extending axons through a single barrier guided by grooves to the axonal compartment. Scale bar = 250μm. B) Diagram depicting the compartment where virus was added (indicated by a red virion), and where sampling occurred (blue background). Titers of WT (strain 17) or Δγ34.5 viruses in the soma compartment 72 hours post-axonal infection with 10^8^ PFU in 129SVEV and STAT1^-/-^ neurons. Cultures were untreated or treated with 12.5U/mL IFNβ in the soma or axon compartment. Dashed line represents the limit of detection. Error bars represent SEM of a minimum of 3 experiments with >2 chambers each. Significance was evaluated by two-way ANOVA where ** p<0.01, ***p<0.001.

To address neuronal IFN signaling in an *in vitro* system which models the *in vivo* route of HSV infection, we utilized Campenot chambers to allow for directional growth of neurons, separating cell body (soma) from axon terminals [33,34]. We modified this system by removing one of the two central barriers to allow for growth of adult TG neurons, which failed to robustly extend neurites through a standard double barrier (Fig 1A). An average of 30.3% (*SD* = 2.25) of the TG neurons extended a network of axons through the single barrier, as judged by the addition of DiI (lipophilic dye) to the axonal compartment. Further characterization of the neuronal subtypes previously shown to be important during HSV infection revealed expected percentages of KH10 and A5 neurons extending axons across the barrier (S1 Fig) [32]. To confirm the barrier integrity of modified Campenot chambers, we added a low molecular weight dextran-conjugated fluorescent protein to the axon compartment of neuron cultures. The low mean fluorescent intensity in the soma compartment indicated that these modified chambers provided a sufficiently tight barrier to diffusion (S1 Fig).

While sensory neurons are capable of signaling IFN, it is unknown whether this can occur specifically at axon terminals to generate an antiviral response. It is likely that IFN is synthesized from an infected mucosal surface, and this secreted IFN has the potential to signal axon terminals of innervating neurons, rendering them resistant to subsequent infection. To address this, IFNβ was added to the axon compartment of wild-type (129SVEV) cultured neurons prior to axonal infection with WT (strain 17) virus, and then viral titers were measured in the soma compartment. Surprisingly, we saw a modest, but significant 4-fold reduction in viral titers in axonal IFNβ-treated compared to untreated neurons. This demonstrated that IFNβ can signal adult sensory neurons via axon terminals (Fig 1B). The HSV protein, ICP34.5, is critical for inhibiting the neuronal antiviral response [19,22] and we therefore hypothesized that ICP34.5 is important for countering the effects of axonal IFN signaling. To test this we treated cultures with IFNβ in the axon compartment and then infected these neurons axonally with a virus lacking ICP34.5 (Δγ34.5). We observed a significant (10-fold) reduction in Δγ34.5 titers recovered at the soma compared to untreated cultures, and compared to WT infected IFNβ treated cultures (*p*<0.0001). This suggests that ICP34.5 may play a role in countering axonal IFNβ signaling. To verify that these effects were dependent upon IFN receptor signaling, we used neurons isolated from isogenic STAT1^-/-^ mice. As expected, the titers of WT and Δγ34.5 viruses were comparable in the presence or absence of IFN, demonstrating that the reductions in titers previously seen were completely STAT1-dependent (Fig 1B).

Having shown that IFNβ can signal via axon terminals, we wished to assess whether IFN treatment of the soma can restrict HSV-1 following infection via the axon. This invokes the *in vivo* scenario whereby IFN produced by a variety of infected cells acts on the soma of TG neurons prior to retrograde transport of HSV-1 from the mucosal surface. In 129SVEV neurons, addition of IFNβ to the soma compartment resulted in a 6-fold reduction of WT and a 65-fold reduction of Δγ34.5 titers compared to untreated cells (Fig 1B). These reductions were completely reversed in neurons isolated from STAT1^-/-^ mice (Fig 1B). Together, these data demonstrate that IFNβ can signal the length of the sensory neuron at both the soma and axon to restrict HSV-1 infection, and that ICP34.5 may counteract this host cell response.

### Axonal IFNβ signaling restricts HSV-1 titers through mechanisms independent of antiviral signaling at the soma

**Fig 2:**
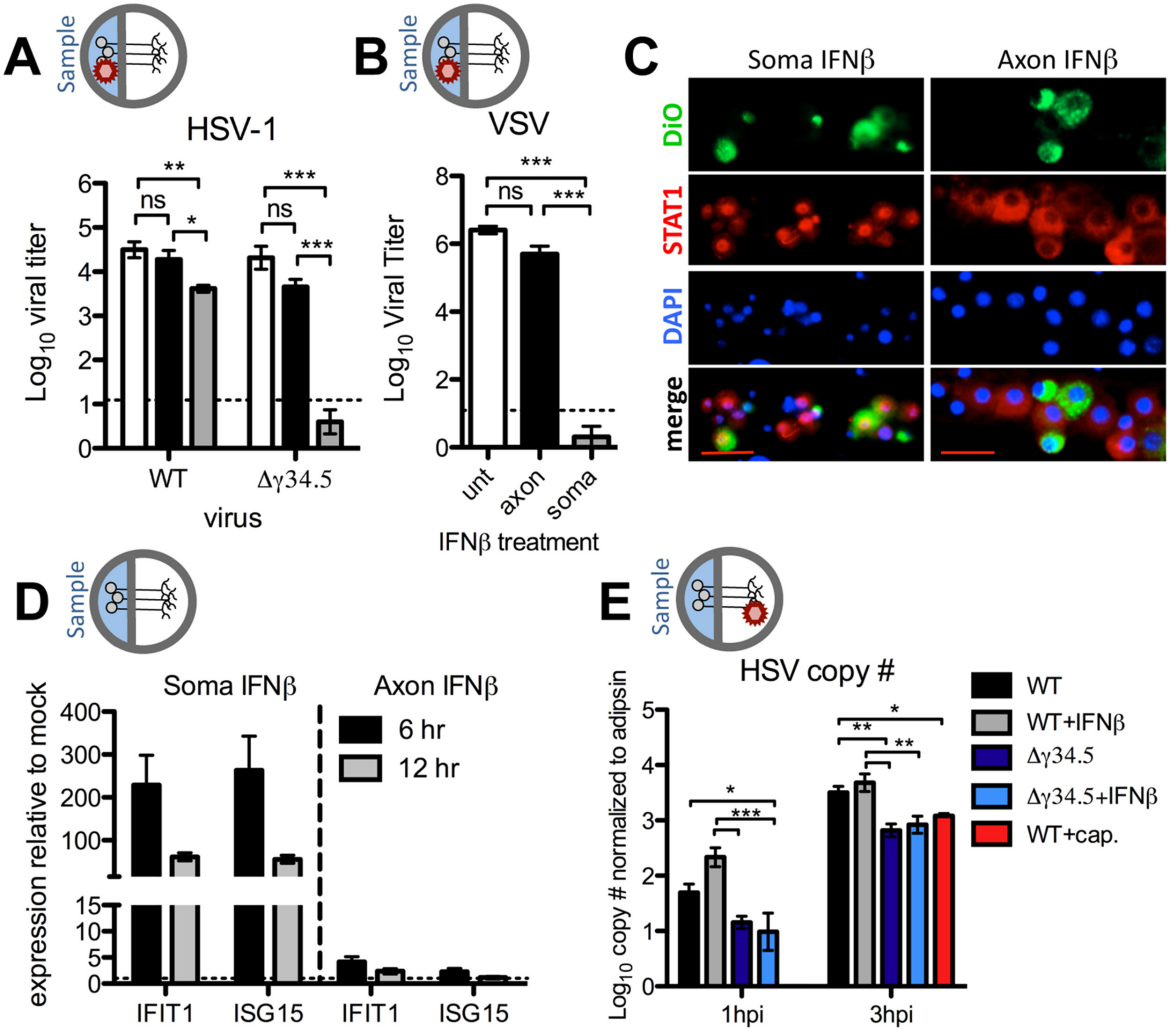
Axonal IFNβ signaling restricts HSV-1 titers through mechanisms independent of establishment of an antiviral state at the soma. A) Titers of WT (strain 17) or Δγ34.5 viruses in the soma compartment of 129SVEV neuron cultures 72 hours post infection via the soma. Cultures were untreated (white bars) or treated with 12.5U/mL IFNβ in the soma (grey bars) or axon compartment (black bars) 18 hours prior to infection. B) Titers of VSV in the soma compartment 24 hours post infection via the soma of 129SVEV neuron cultures. Cultures were treated with IFNβ as in (A). C) Representative image of immunofluorescence staining for STAT1 (red) in chambers treated with IFNβ at the soma for 3 hours (left) or axon for 3, 5, 7 or 16 hours (right). Cells were counterstained for nuclei (DAPI, blue). DiO (green) was added to the axon compartment, thereby labeling neurons that extended axons through the central barrier. Scale bar = 50μm. D) Expression of IFIT1 and ISG15 transcripts in the soma compartment of chambers treated with IFNβ for 6 or 12 hours at the soma or axon. Error bars represent SEM of a minimum of 4 samples over 2 experiments. E) HSV genome copy number of WT (strain 17) or Δγ34.5 virus measured from the soma compartment of axonally infected 129SVEV neuron cultures at 1hpi and 3hpi. Cultures were treated with 100μM ACV, and with 12.5 U/mL IFNβ in the axon compartment for 18 hours, or 10μM capsaicin (red bar) in both compartments for 0.5 hour prior to infection with 10^8^ PFU. Error bars represent SEM of a minimum of 3 repeats with >3 chambers each. Two-way ANOVA was performed where *p<0.05, ** p<0.01, ***p<0.001.

We wished to address whether establishment of an antiviral state was responsible for restriction of viral titers following axonal IFNβ treatment of chamber cultures. To test this, we added IFNβ to the axon compartment, and the soma compartment as a control. We then infected the soma compartment with WT, or the IFN-sensitive Δγ34.5 virus. Therefore, if axonal IFNβ signaling induces an antiviral state at the soma, we would expect reduced Δγ34.5 viral titers after soma infection. Consistent with our previously published results, IFNβ added to the soma significantly (700-fold) reduced titers of Δγ34.5 virus (Fig 2A). Unexpectedly, there was also a small, but significant (9-fold) reduction in WT virus titers, likely reflecting differences between coverslip and Campenot chamber cultures [22]. Most notably, however, addition of axonal IFNβ did not change soma-derived titers of either WT or Δγ34.5, demonstrating that axonal IFNβ signaling may not lead to establishment of a conventional antiviral state at the soma (Fig 2A).

HSV encodes for several proteins besides ICP34.5 that counteract an IFN response [11]. This raises the caveat that the lack of an antiviral state at the soma is due to IFN disruption by viral proteins other than ICP34.5. We therefore utilized VSV, a highly IFN-sensitive virus, and consistent with the data for Δγ34.5, we observed no significant decrease in VSV replication upon axonal IFNβ treatment at 24hpi (Fig 2B). This observation held true when higher IFNβ concentrations were employed (100U/mL, S2 Fig). In contrast, and as expected, we observed a dramatic reduction in VSV replication upon IFNβ treatment of the soma at 24hpi (Fig 2B).

To further investigate axonal IFN signaling, STAT1 nuclear relocalization in neurons exposed to axonal IFN was examined by immunofluorescence. DiO was added to the axonal chamber to label neurons with axons that extended through the central barrier. STAT1 localization remained cytoplasmic at all timepoints tested following axonal IFN treatment (Fig 2C). In contrast, we observed robust nuclear STAT1 relocalization in neurons exposed to soma IFN, consistent with previous data [22]. We next measured transcript levels of two interferon stimulated genes (ISGs), IFIT1 and ISG15, after soma or axonal IFN treatment. Consistent with our titer and immunofluorescence data, we observed minimal upregulation of either IFIT1 or ISG15 at both 6 and 12 hours post-axonal IFN treatment (Fig 2D). In contrast, we observed a large upregulation of both ISGs after soma IFN treatment. Together these data demonstrate that axonal IFNβ signaling restricts yields of WT and Δγ34.5 virus after axonal infection by a mechanism independent of antiviral signaling at the soma.

A previous study demonstrated that IFN signaling can restrict trafficking of poliovirus to the CNS [35]. Our data are consistent with this idea in that axonal IFNβ signaling may affect retrograde viral transport, which in turn results in reduced HSV titers. To address this, we infected neurons via the axons and then measured the number of incoming viral genomes at the soma in the presence or absence of axonal IFNβ. Unexpectedly, we found no significant change in the number of viral genomes accumulating in the soma compartment of untreated or IFNβ treated cultures (Fig 2E). Furthermore, treatment of neurons with capsaicin, previously shown to reduce retrograde transport [36], resulted in decreased accumulation of viral genomes, validating this viral capsid trafficking assay. We additionally observed no difference in genome copy number when a higher concentration of IFN (100U/mL) and lower inoculum of virus (10^6^ PFU) was employed (S3 Fig). Interestingly, we observed a significant reduction in the number of Δγ34.5 genomes relative to WT, regardless of IFNβ treatment, suggesting that ICP34.5 affects retrograde transport. It is therefore possible that the additional restriction of Δγ34.5 titers upon axonal infection (Fig 1B) is due to an inherent defect in retrograde transport of Δγ34.5 mutants. Together, these data demonstrate that IFNβ acts on neurons at both the cell body and the axon to control HSV-1 through distinct STAT1-dependent mechanisms (S4 Fig).

### Validation of conditional STAT1 knockout mice

Having shown that IFNβ signaling in TG neurons is important for restricting HSV-1 replication *in vitro*, we wished to address its role *in vivo*. Previous work infecting IFN-signaling null mice resulted in generalized lethal disease with viral spread to multiple organs [7,9,37]. Also, conditional knockout mice lacking IFNα responses in neural tissue are more susceptible to VSV and Rabies virus, suggesting a role for neuronal IFN signaling in controlling these viral infections [38,39]. To address neural IFN signaling more generally in the context of HSV-1 infection, mice with Cre recombinase under the neural-specific Nestin promoter were crossed with STAT1 floxed mice. This thereby generated a new mouse model (Stat1^N-/-^) with intact IFN signaling in all tissues except neuroectoderm-derived cells, (e.g. PNS and CNS neurons, PNS satellite glial cells, and astrocytes). Littermate control mice (Stat1^fl/fl^) were used for all experiments, and Stat1^N-/-^ and Stat1^fl/fl^ mice were equally viable. While the background strain of the conditional knockout mice (C57/Bl6) differs from that of the neurons used for *in vitro* studies (129SVEV), we have shown that neurons derived from these strains support equivalent rates of HSV replication [22].

**Fig 3:**
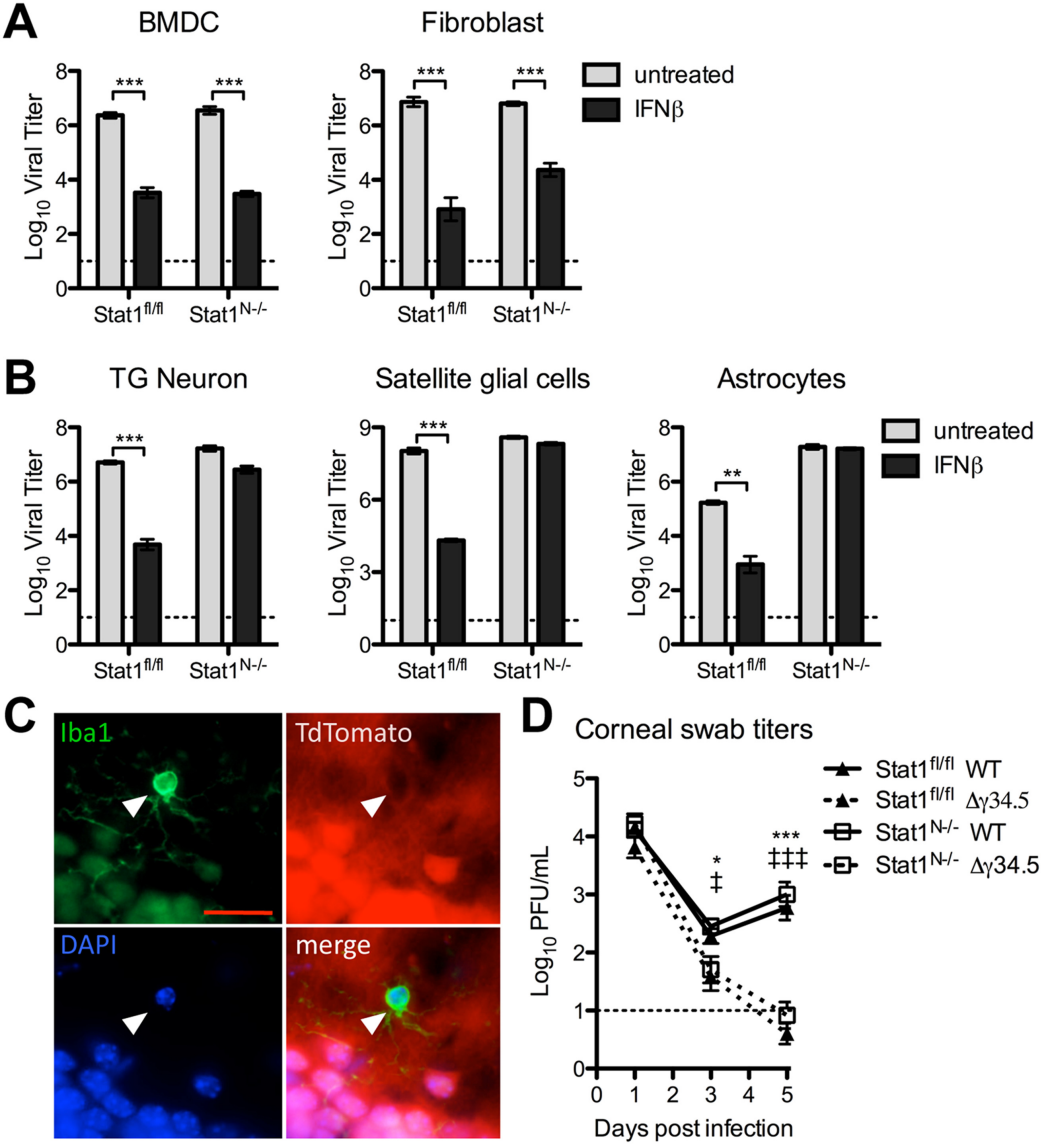
Validation of Stat1^N-/-^ mouse. Titers of VSV in BMDCs or fibroblasts (A) or TG neurons, satellite glial cells, or astrocytes (B) isolated from Stat1^N-/-^ or Stat1^fl/fl^ mice. Cells were untreated or treated with 12.5 units/mL IFNβ and titers were measured at 24hpi. Error bars represent SEM of at least two experiments with ≥2 samples each. C) Immunofluorescence staining of brain tissue from 3 week old progeny from a cross of TdTomato reporter and Nestin Cre mice. TdTomato fluorescence indicates cells that express or expressed CRE recombinase. White arrow marks Iba1+ microglia (green). Cells were counterstained for DAPI (blue). Scale bar = 20μm. D) Viral titers from corneal eye swabs of Stat1^N-/-^ or Stat1^fl/fl^ mice infected via corneal scarification with 2 x 10^6^ PFU/eye WT (stain 17) or Δγ34.5 virus. Dashed line represents the limit of detection, and error bars represent SEM of a minimum of 7 mice, over at least 2 experiments. Two-way ANOVA with Bonferroni correction was performed where one symbol indicates p<0.05, and three symbols indicate p<0.001. Unless noted with brackets, * indicates significant differences between Stat1^N-/-^ WT and Stat1^N-/-^ Δγ34.5; and ‡ between Stat1^fl/fl^ WT and Stat1^fl/fl^ Δγ34.5.

To verify the IFN signaling status of neural and non-neural tissues, we cultured TG neurons, fibroblasts, bone marrow-derived dendritic cells (BMDCs), satellite glial cells (SGCs) and astrocytes isolated from naive Stat1^N-/-^ and Stat1^fl/fl^ mice. Cells were treated with IFNβ and replication of VSV was measured. As expected, fibroblasts and BMDCs isolated from both Stat1^N-/-^ and Stat1^fl/fl^ mice restricted VSV replication when treated with IFNβ (Fig 3A). In contrast, TG neurons, SGCs and astrocytes isolated from Stat1^N-/-^ mice were significantly less able to control VSV replication compared to Stat1^fl/fl^ neurons (Fig 3B). These results are consistent with previously published reports of Nestin expression [40]. Through crossing the Nestin-Cre mouse with a reporter mouse expressing TdTomato following Cre-mediated recombination, we additionally verified that microglia of the CNS do not express TdTomato and are thus STAT1 sufficient in our system (Fig 3C).

These results demonstrated that IFN signaling was intact in non-neural tissues of Stat1^N-/-^ mice, and predicted that IFN signaling should control HSV replication in non-neuronal tissues such as the cornea. To examine this, we infected mice via the cornea with WT or Δγ34.5 virus and measured corneal swab titers (Fig 3D). As expected, we found no difference in WT viral titers, and Δγ34.5 was equally and highly attenuated in corneas of Stat1^fl/fl^ and Stat1^N-/-^ mice (Fig 3D).

### Neural Stat1 expression is critical for controlling HSV-1 replication *in vivo*

**Fig 4:**
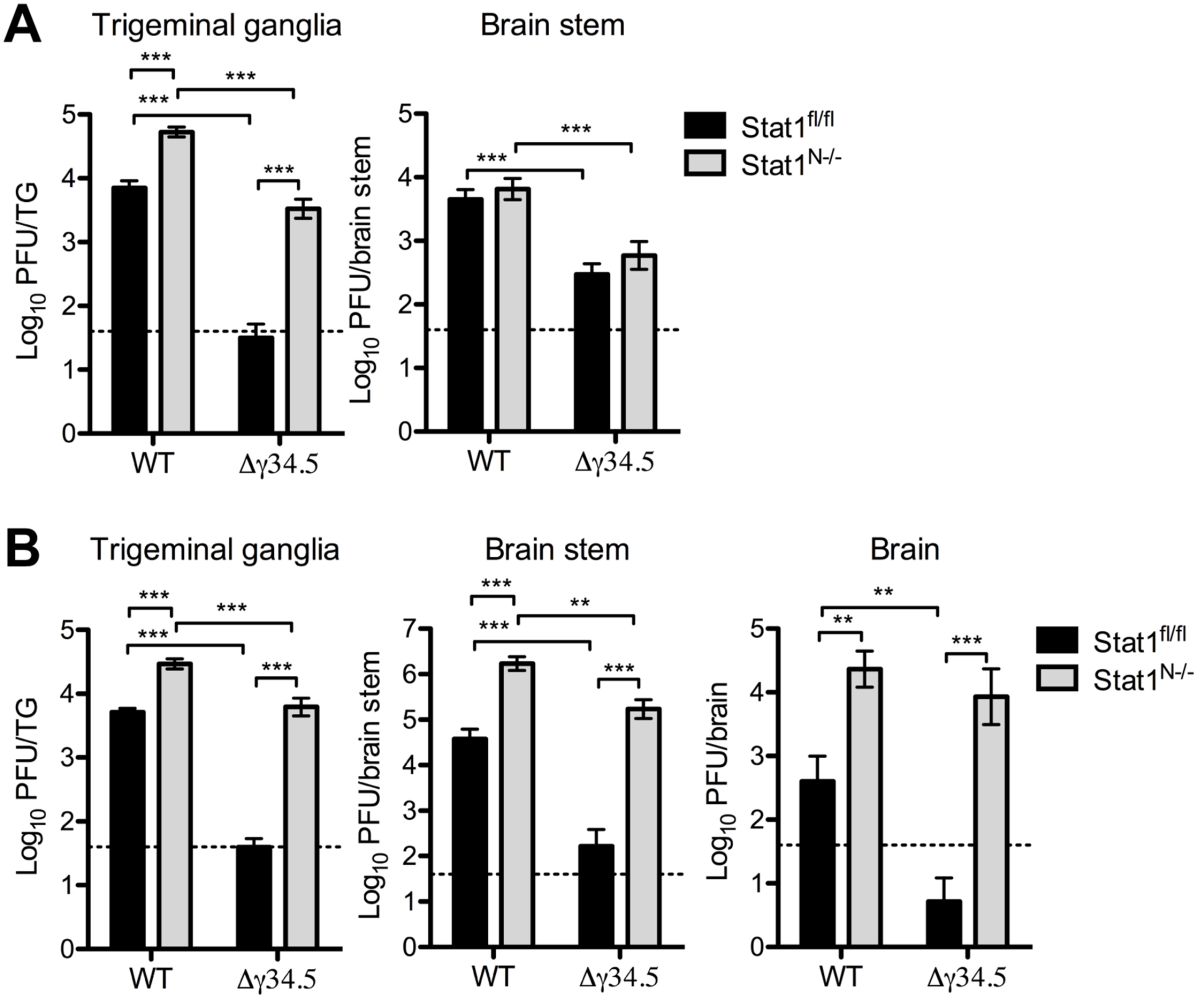
Neural STAT1 expression is critical for controlling HSV-1 replication *in vivo*. Viral titers in the TG, brain stem and brain of Stat1^N-/-^ or Stat1^fl/fl^ mice infected via corneal scarification with 2 x 10^6^ PFU/eye WT (strain 17) or Δγ34.5 virus. Viral titers were measured at 3 dpi (A) and 5 dpi (B). Dashed lines represent the limit of detection. Error bars represent SEM of a minimum of 13 mice total over 2 experiments. Two-way ANOVA was performed where ** p<0.01, ***p<0.001.

To examine virus replication in the nervous system, we next infected Stat1^N-/-^ and Stat1^fl/fl^ mice corneally with WT or Δγ34.5 viruses then measured titers in the TG, brain stem and brain. There was a significant increase in WT titers in the TGs of infected Stat1^N-/-^ compared to Stat1^fl/fl^ mice (Fig 4A and 4B). Additionally, Δγ34.5 titers in the TGs of Stat1^N-/-^ mice were significantly increased by ~100-fold on both days almost achieving the titers of WT virus. On day 3, low levels of virus were observed in the brain stem and notably there were no differences in the titers between Stat1^N-/-^ and Stat1^fl/fl^ mice (Fig 4A). No virus was detected in the brain at this timepoint. On day 5, however, significant increases in WT viral titers were observed in brain stems and brains of Stat1^N-/-^ mice compared to Stat1^fl/fl^ (Fig 4B). Moreover, we saw large increases in Δγ34.5 titers in the brain stems and brains of Stat1^N-/-^ mice, with low titers in littermate controls (Fig 4B). Together, these data show that neuronal STAT1 expression is critical for controlling HSV-1 replication in nervous tissue and that ICP34.5 counters this STAT1-driven response.

### Neural STAT1-deficiency affects viral tropism in the trigeminal ganglia

**Fig 5:**
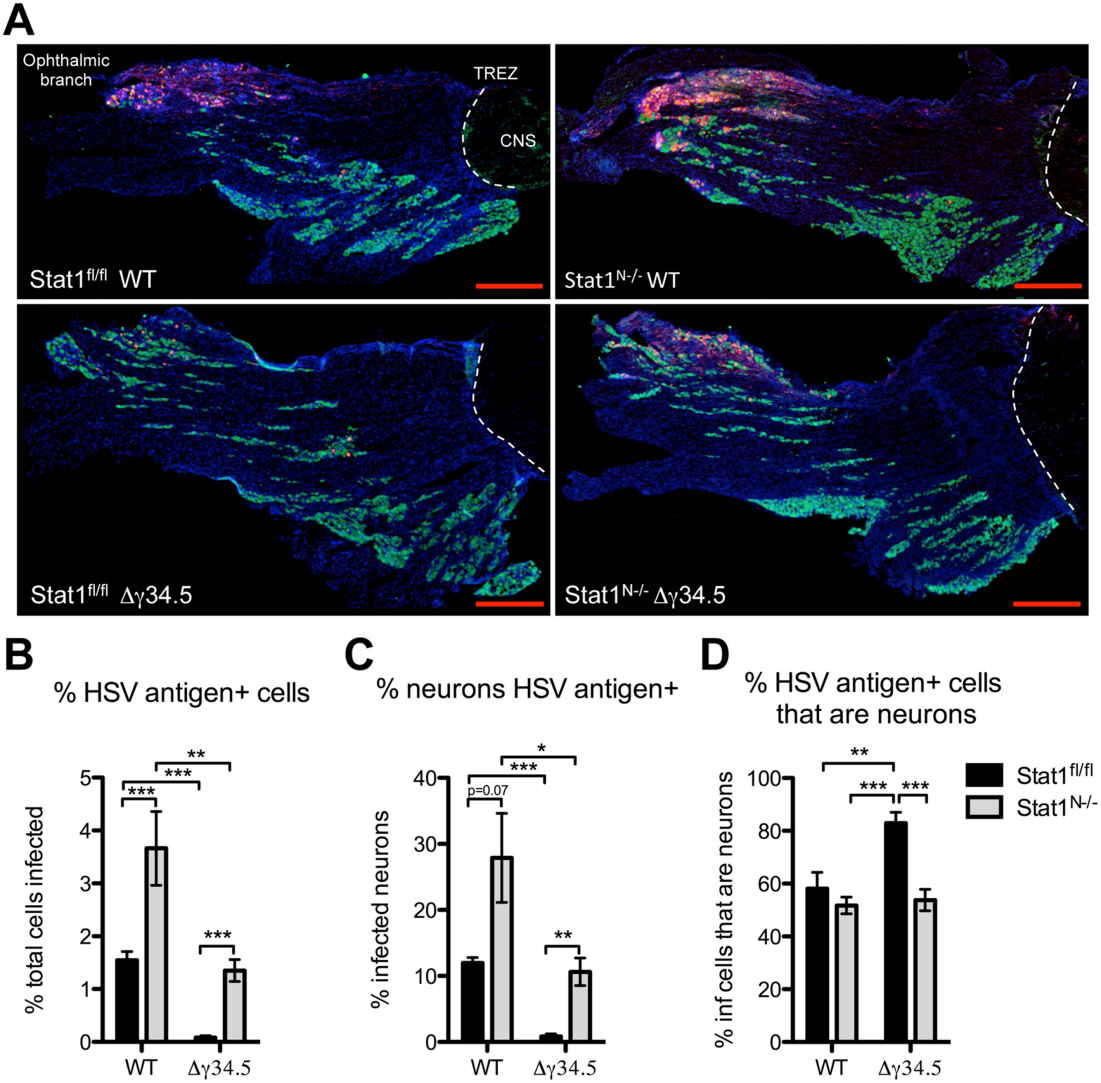
Neural deletion of STAT1 affects viral tropism in the trigeminal ganglia. A) Immunofluorescence of TG sections from Stat1^N-/-^ or Stat1^fl/fl^ mice 5 days post infection with 2 x 10^6^ PFU/eye WT (strain 17) or Δγ34.5 virus via the cornea. Tissue sections show immunostaining for HSV antigen (red), the neuronal marker NeuN (green), and nuclei (DAPI, blue). The ophthalmic branch of the TG is indicated and white dotted lines mark the trigeminal root entry zone (TREZ) delineating the boundary between PNS and CNS. Scale bar = 500 μm. B) Quantification of the percentage of total cells that are HSV antigen-positive. C) Quantification of the percent of total neurons that are HSV antigen-positive. D) Quantification of the percent of HSV antigen-positive cells that are neurons. Quantification was done using ImageJ/Fuji. Individual data points represent the average of >5 tissue slices per TG with a minimum of 7 TGs over 3 experiments. Student’s t-test was performed where *p<0.05, ** p<0.01, ***p<0.001.

IFN signaling is important for restricting tropism of neurotropic viruses [25,37]. We therefore wished to address whether neuronal IFN signaling restricts HSV-1 infection to sensory neurons thereby affecting cellular tropism within the TG. To test this, we examined the colocalization of neurons (green), and HSV antigen (red) in the TG (Fig 5A). Quantification of virus antigen-positive cells showed a close correlation with the titer data (Fig 4) with significantly more cells infected in Stat1^N-/-^ mice compared to Stat1^fl/fl^ in both WT and Δγ34.5 infections (Fig 5B). Similarly, more neurons were infected in Stat1^N-/-^ mice compared to Stat1^fl/fl^ in both WT and Δγ34.5 infections (Fig 5C).

As a measure of virus tropism we next quantified the number of infected neurons from each group and expressed this as a percentage of the total number of infected cells. In Δγ34.5-infected Stat1^fl/fl^ mice, >80% of infected cells were neurons, with relatively few infected non-neuronal cells (Fig 5D). Notably, however, the percentage of Δγ34.5 infected cells that were neurons in Stat1^N-/-^ mice was significantly lower than that seen in Stat1^fl/fl^ mice. This suggests that IFN signaling in the projecting infected neuron prevents spread of incoming Δγ34.5 in the TG (Fig 5D). Additionally, there were significantly more infected non-neuronal cells in WT compared to Δγ34.5 infected Stat1^fl/fl^ mice. Upon closer examination, we further observed that SGCs, which surround the neuronal cell body, become infected in Stat1^N-/-^ and st17 infected Stat1^fl/fl^ mice (S5 Fig). In contrast, this was not observed in Δγ34.5 infected Stat1^fl/fl^ mice (S5 Fig). These data suggest that ICP34.5 promotes spread to non-neuronal cells in the TG, even in the presence of intact IFN signaling (Fig 5D).

### Viral zosteriform spread and pathogenesis in non-neuronal tissues of Stat1^N-/-^ mice

**Fig 6:**
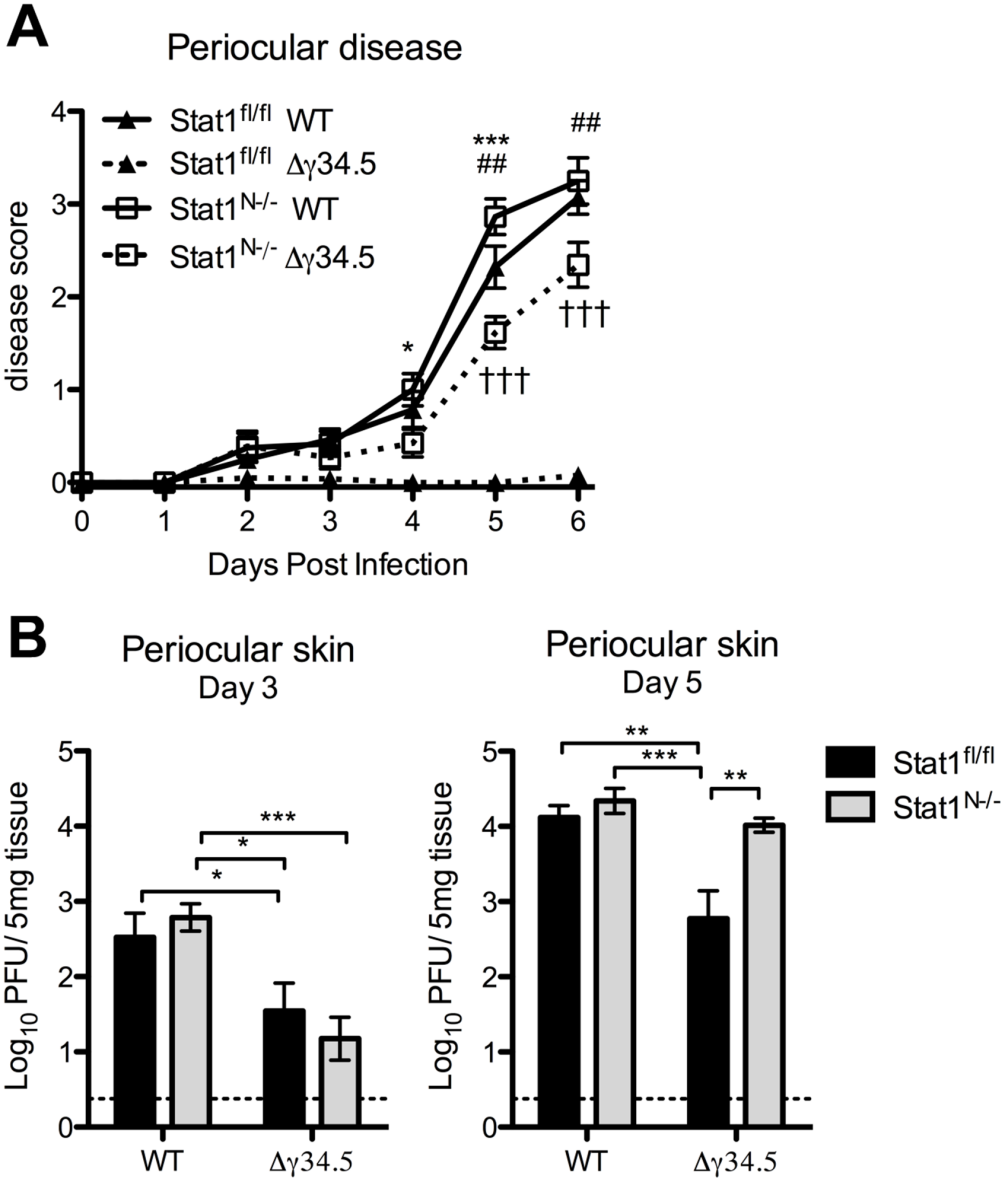
Viral zosteriform spread and pathogenesis in non-neuronal tissues of Stat1^N-/-^ mice. A) Periocular disease in Stat1^N-/-^ or Stat1^fl/fl^ mice infected via the cornea with 2 x 10^6^ PFU/eye WT (strain 17) or Δγ34.5 virus. Disease scoring was based on a 1–4 scale. B) Titers of periocular skin from Stat1^N-/-^ or Stat1^fl/fl^ mice infected as in (A). Data points represent the average of 2 skin punch titers from each eye. Dashed lines delineate the limit of detection, and error bars represent SEM of a minimum 12 mice, over at least 2 experiments. Two-way ANOVA was performed where one symbol indicates p<0.05, two symbols p<0.01, and three symbols p<0.001. Unless noted with brackets, * indicates significant differences between Stat1^N-/-^ WT and Stat1^N-/-^ Δγ34.5; # between Stat1^fl/fl^ WT and Stat1^N-/-^ Δγ34.5; and † between Stat1^fl/fl^ Δγ34.5 and Stat1^N-/-^ Δγ34.5.

Zosteriform spread involves the retrograde transport of virus from infected mucosae via the peripheral nerves to the TG, followed by anterograde transport to innervated tissue distal to the site of initial infection [1]. Following corneal infection in the mouse and in humans, periocular skin infection and disease are likely a consequence of zosteriform spread of the virus rather than direct spread from the cornea, which is dependent on robust replication in the innervating TG [41]. Based on this model, our observed pattern of viral titers in the TG predicts that WT virus would cause significantly more periocular infection and disease than Δγ34.5 in Stat1^fl/fl^ mice, and this should be normalized in Stat1^N-/-^ mice, despite the presence of STAT1-dependent responses in the skin. Consistent with this hypothesis, we observed significantly more periocular disease in Stat1^fl/fl^ mice infected with WT virus compared to Δγ34.5 (Fig 6A). Furthermore, there was significantly more disease in Δγ34.5 infected Stat1^N-/-^ mice compared to Stat1^fl/fl^ mice, with disease levels approaching those seen in WT virus-infected mice. While this significantly increased and overt disease was in contrast to the low levels of Δγ34.5 virus (<10pfu) in corneal swabs of the Stat1^N-/-^ mice (Fig 3B), it correlated with a significant increase in skin titers on day 5 (Fig 6B). These data therefore suggest that the lack of neural IFN-signaling causing increased replication in the TG of Stat1^N-/-^ mice promotes periocular disease due to zosteriform spread of HSV-1. These data therefore further validate the zosteriform spread model and the phenotype of Stat1^N-/-^ mice [41].

### Neural STAT1 expression is required for host survival

**Fig 7:**
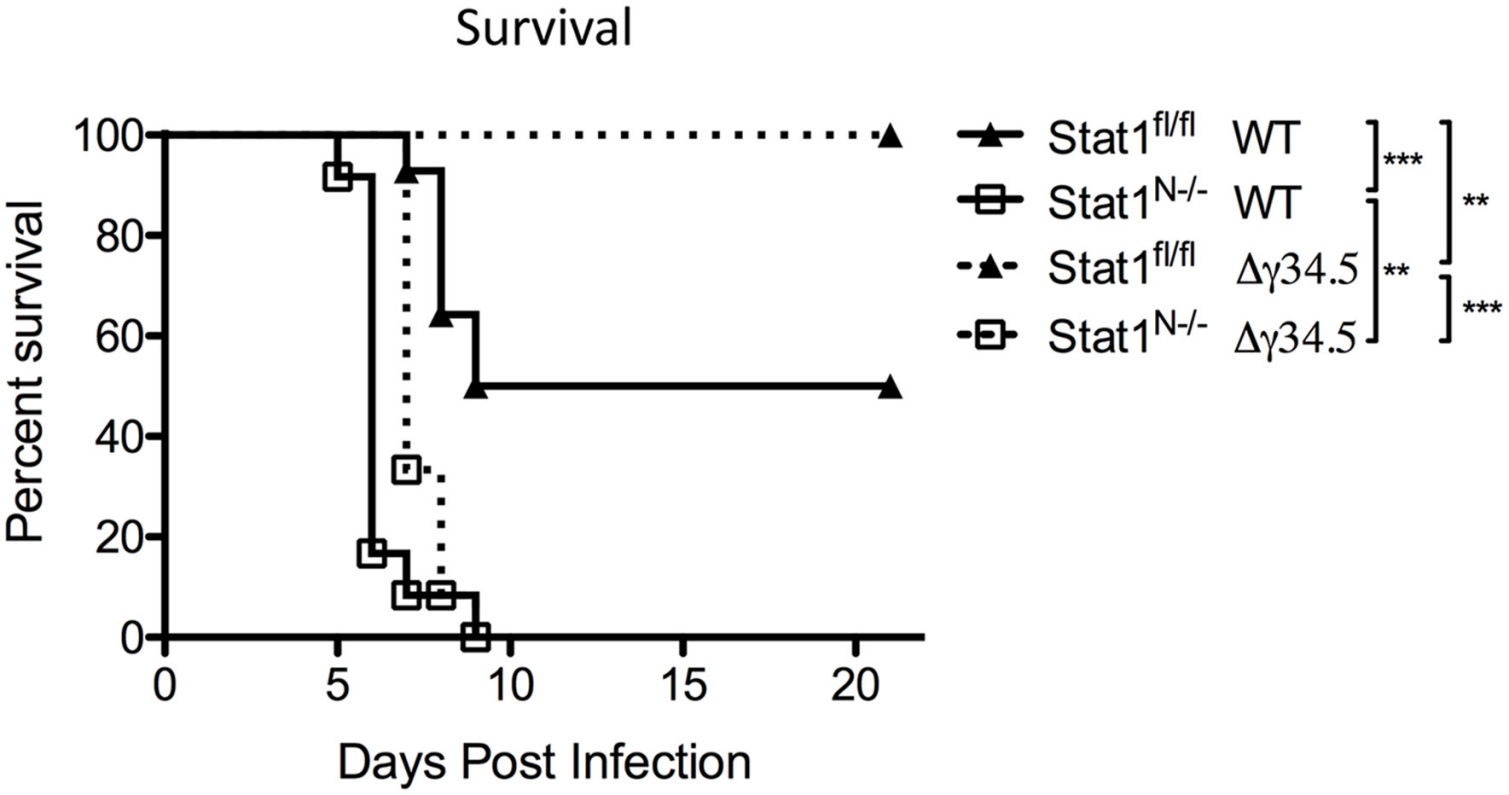
Neural STAT1 expression is required for host survival. Survival of Stat1^N-/-^ or Stat1^fl/fl^ mice infected via the cornea with 2 x 10^6^ PFU/eye strain 17 (st17) or Δγ34.5 virus. Mice were monitored over time and were euthanized upon reaching end-point criteria, here referred to as survival. Data represent two independent experiments where Stat1^fl/fl^ WT n = 14 mice, Stat1^N-/-^ WT n = 12, Stat1^fl/fl^ Δγ34.5 n = 13 and Stat1^N-/-^ Δγ34.5 n = 12 mice. Survival was plotted on a Kaplan-Meier curve and statistics performed with Log-rank test where ** p<0.01, ***p<0.001.

We next tested how neuronal expression of STAT1 impacts host survival following peripheral (corneal) challenge. Approximately 50% of Stat1^fl/fl^ mice infected with WT virus succumb to infection over a 21-day timecourse (Fig 7). In contrast, 100% of Stat1^N-/-^ mice infected with WT virus succumbed rapidly to infection by day 9 (Fig 7). As expected, 100% of Stat1^fl/fl^ mice survived infection with Δγ34.5, but 100% of Stat1^N-/-^ mice infected with Δγ34.5 died, demonstrating that neural STAT1 deletion restores virulence to Δγ34.5 (Fig 7). While these mice died within same 9-day window seen with WT virus, the timecourse was slower (*p*<0.01). Importantly, these data show that non-neural STAT1 expression alone is insufficient to control virulence of WT and Δγ34.5 virus.

## Discussion

Loss of IFN signaling results in significantly increased HSV-1 pathogenesis and mortality in humans and mice but the specific role of IFN signaling in neurons is unclear [6,7,9]. Here, we demonstrate the importance of a functional neuronal IFN response in resistance to HSV-1 replication and pathogenesis in mice with a neural-specific deletion of STAT1. STAT1 is a key transcription factor that is downstream of multiple interferon receptors that include type I (α and β), II (γ) and III (λ) [8]. We demonstrated *in vitro* that IFNβ is able to restrict neuronal HSV-1 replication, but it is possible that multiple IFNs are acting upon neurons *in vivo*. Studies examining the capacity of cultured TG neurons to upregulate an effective antiviral response revealed that IFNβ plays a predominant role in neuronal antiviral signaling. That said, TG neuron cultures also have the ability to respond moderately to IFNγ, and modestly to IFNλ, as demonstrated by STAT1 nuclear localization and inhibition of VSV replication (S6 Fig). Consistent with a potential role for IFNγ signaling, functional IFNγ receptors are present at the axon terminals of peripheral neurons [42] and IFNγ can reduce reactivation of HSV-1 from explanted TGs [43]. Additionally, neuronal IFNλ restricts HSV-1 replication *in vitro* [44], and IFNλ treatment can exert protective effects *in vivo* against HSV-2 disease [45]. It will thus be important to further investigate the roles of type II and III IFN both *in vitro* and *in vivo* on neuronal HSV replication and viral pathogenesis.

Type I IFN signaling is a determinant of tissue tropism of neuroinvasive viruses such as West Nile virus, poliovirus, and HSV-1 [25,37,46]. It is unclear, however, how IFN signaling impacts this tropism on a cellular level. The proportion of non-neurons that are infected was significantly higher in the TGs of Δγ34.5 infected mice lacking neural STAT1 expression, revealing a role for neural IFN signaling in restricting HSV tropism to sensory neurons and counteraction of this by HSV. Furthermore, we demonstrated that there is a host-pathogen balance in determining cell tropism, as ICP34.5 effectively counteracts this IFN signaling, thereby promoting viral spread within the TG. These data are consistent with previous work showing that the absence of TLR3 signaling changes the tropism of HSV-2 such that it infects astrocytes rather than neurons of dorsal root ganglia [47]. Together these data show that neuronal innate responses control viral dissemination and cell-type predilection in the sensory ganglia.

Altered tropism in the TG and subsequent anterograde viral spread may impact dissemination to the periocular skin. Murine models of HSV-1 infection, and clinical studies in humans have demonstrated zosteriform spread whereby the virus infects sites distal from the initial site of infection through anterograde trafficking from the sensory ganglia [1,41]. Given the significant attenuation of Δγ34.5 in the presence of IFN responses, and restoration of its replication in the absence of IFN responses, infection of Stat1^N-/-^ mice with Δγ34.5 provided a unique insight into zosteriform spread and periocular disease. While corneal titers of Δγ34.5 were comparably low in both Stat1^N-/-^ and control mice, overt periocular disease was only apparent in Stat1^N-/-^ mice. The significant increase in periocular disease correlated with the high viral load in the TG and periocular skin of Δγ34.5 infected Stat1^N-/-^ mice compared to littermate controls. These data validate previous findings that zosteriform spread from the TG to the periocular skin is the cause of periocular disease in corneal HSV infection models [41]. This is also consistent with previous studies of corneal and alternate routes of HSV-1 infection in mice, and clinical findings of periocular lesions during herpes keratitis [41,48–50]

Our *in vitro* data support a role for neuronal STAT1-dependent IFNβ signaling at both the soma and axon in controlling HSV-1 following axonal infection. In contrast, there was no evidence that this STAT1-dependent signaling via the axon could control HSV-1 following infection of the soma or lead to a significant upregulation of ISGs. We were also unable to detect STAT1 in the nucleus of neurons post-axonal IFN treatment. This was an unexpected finding given that, canonically, STAT1 mediates signaling via translocation from the cytoplasm to the nucleus. These data suggest that STAT1 has a non-canonical function that is disrupting a process important to the viral life cycle prior to replication at the soma. An example of such non-canonical STAT signaling has been described for STAT3 which binds to stathmin, thereby stabilizing microtubules [51,52]. While we did not detect a change in DNA-containing capsid trafficking, STAT1 signaling may interfere with retrograde transport of tegument proteins which would, in turn, result in restricted viral replication [2]. These data also suggest that use of topical IFN may be an effective therapy for ocular or oral HSV lesions by stimulating neuronal antiviral signaling at axon terminals innervating the site of infection. Indeed clinical studies on use of topical IFN in conjunction with antivirals were promising [53].

Another unexpected finding was the significant reduction in the number of Δγ34.5 genomes reaching the soma after axonal infection, independent of IFN treatment, suggesting a novel role for ICP34.5 in regulating retrograde trafficking or entry. Some studies have suggested the presence of ICP34.5 in the viral tegument, although at low abundance relative to other proteins [54]. It is formally possible, therefore, that ICP34.5 derived from entering virions could be directly exerting an effect upon neurons. It is more probable, however, that in the absence of ICP34.5, expression of structural proteins that are important in trafficking or entry are reduced in the ICP34.5 mutant [55]. This reduced trafficking or entry of ICP34.5-deficient viruses in neurons may be particularly important and useful in enhancing safety during their potential use for oncolytic therapy of glioblastoma [17].

Our studies demonstrate an important role for ICP34.5 in combating neuronal IFN signaling. While ICP34.5 has multiple functional domains that can counteract IFN signaling [12–14], it also contains a Beclin binding domain (bbd) which can interfere with neuronal autophagy, an important response in combating HSV-1 [16,21]. We add to these findings and demonstrate that neuronal IFN signaling is also critical for controlling HSV-1 infection. It is likely that autophagic and IFN-signaling pathways synergize in sensory neurons. Indeed, there is evidence supporting a role for IFN signaling in the upregulation of autophagy [22,56]. Given this, the absence of neuronal IFN signaling combined with a dysregulation of autophagy may account for the dramatic increase in Δγ34.5 virulence in Stat1^N-/-^ mice. As such, it will be important to examine autophagy in IFN signaling deficient neurons *in vitro* and in Stat1^N-/-^ mice.

The degree to which neuronal IFN signaling is critical for host survival is particularly striking. Stat1^N-/-^ mice were markedly susceptible to infection, and the normally attenuated Δγ34.5 virus was nearly restored to full virulence in these mice. While it is possible that the effects seen *in vivo* were exacerbated by the additional loss of IFN signaling in SGCs and astrocytes, these data importantly showed that IFN signaling of the immune system and of peripheral tissues is less critical than IFN-driven innate responses in neural tissues. The data additionally demonstrated that ICP34.5 is critical for viral resistance to neural IFN responses, affirming the role of ICP34.5 as a specific neurovirulence factor. Ultimately, our *in vitro* and *in vivo* results demonstrate a requirement for neuronal STAT1 signaling in controlling HSV-1 pathogenesis, and the new models generated herein will prove useful for subsequent studies on the pathogenesis of HSV and other clinically important neuroinvasive pathogens.

## Materials and Methods

### Neuron and satellite gilal cell isolation and culture

Trigeminal ganglia (TG) neurons were isolated as described previously with some modifications [22,32]. Briefly, 6–10 week old mice were transcardially perfused, TGs harvested and digested in papain (Worthington) followed by collagenase type II (Invitrogen) and neutral protease (Worthington). TGs were then triturated and the resulting homogenate was spun over a four-layer Optiprep (Sigma) density gradient and two bands of lower density were collected and washed three times. Neurons were cultured in NB-A complete media for ≥ 3 days prior to use. NB-A complete media consisted of Neurobasal-A, 2% SM1, 1% GlutaMAX (Invitrogen), 1% penn/strep, 50ng/mL Neurturin (R&D Systems), 50ng/mL neuronal growth factor (NGF, Invitrogen), 50ng/mL glial derived neurotrophic factor (GDNF, R&D Systems), and 60μM FUDR. For satellite glial cell culture, cells resulting from density gradient spin were plated in 24-well tissue culture plates in DMEM (HyClone) with 10% FBS, 1% GlutaMAX (Invitrogen) and 1% penn/strep. Cells were trpysinized once prior to use, removing any contaminating neurons. Cells were infected at an MOI of 20.

### Modified Campenot chambers

20mm CAMP320 chambers (Tyler Research) were modified by removing one internal barrier. Chambers were assembled as described previously [34]. Briefly, vacuum grease was applied to one side of the modified chamber and mounted onto PDL/laminin-coated dishes with 16 parallel grooves etched into the bottom spanning the central barrier and overlayed with 1% methylcellulose in NB-A complete media (described above). Neurons were cultured for 2 weeks and assessed prior use for axonal growth. They were then infected either via the axonal compartment with 10^8^ plaque-forming units (PFU) HSV (MOI 8,300) or via the soma compartment with 24,000 PFU HSV (MOI 2) or 600 PFU VSV (MOI 0.5). The high MOI used for axonal infection was empirically determined to deliver approximately equivalent genome copies as soma infection and is consistent with the literature utilizing such compartmentalized chambers [57]. When indicated, cells were treated with 12.5 units/mL IFNβ (PBL Interferon source) 18 hours prior to infection and maintained throughout. Viral titers were assessed via plaque assay on Vero cells as described previously [58]. For selective labeling of neurons extending axons through the central barrier, the lipophilic dye DiI or DiO (Invitrogen) was added to the axonal compartment at 5μl/mL.

### Chamber barrier integrity

Barrier integrity was assessed in chambers with 2-week old neuron cultures. Dextran-fluorescein conjugated dye (MW = 10,000, Invitrogen) was added to the axonal compartment at 0.2mg/mL and the mean fluorescent intensity (MFI) of supernatant from both compartments was measured after 72 hours on a Zeiss Axio Observer Z1 inverted microscope using Zen software.

### Bone marrow-derived dendritic cells (BMDC) isolation and culture

BMDCs were isolated and cultured as described previously [59]. Briefly, femurs were removed from mice that had been lightly perfused for TG neuronal isolation, flushed and filtered through a 100μM mesh. Cells were seeded at a density of 3 million cells per well and differentiated through culture with RPMI-1640 (HyClone) 1% sodium pyruvate (HyClone), 10% fetal bovine serum (FBS- Atlanta Biologicals), 0.5% penn/strep, 1% L-glutamine (HyClone) and 15% granulocyte-macrophage colony stimulating factor (GM-CSF). Cells were infected at an MOI of 0.1.

### Fibroblast isolation and culture

Fibroblasts from adult mice were obtained through ear clippings and subsequently minced and digested in 1000U/mL collagenase Type II (Invitrogen) followed by 0.05% trypsin (Cellgro). Resulting cell lysate was triturated and plated in 6 well plates in DMEM (HyClone) with 10% FBS, 1% non-essential amino acids, 1% GlutaMAX (Invitrogen) and 1% penn/strep. Cells were infected at an MOI of 0.5.

### Astrocyte isolation and culture

Astrocytes were isolated as previously described [60]. Briefly, cortical hemispheres of p3 mice were obtained and the meninges were removed. Tissue was minced and incubated with 0.1% trypsin for 30 mins. Resulting cell lysate was triturated and plated in T25 flasks in DMEM (HyClone) with 10% FBS, 1% GlutaMAX (Invitrogen) and 1% penn/strep. After 2 weeks of culture, flasks were mechanically shaken to remove microglia. Remaining cells were trypsinized and seeded in 24 well plates. Cells were infected at an MOI of 0.5.

### Viruses and mice

Strains 17 syn+, and the ICP34.5-null mutant on the strain 17 (WT) background, Δγ34.5 were made as previously described [61,62]. Viral stocks were grown on Vero cells as described previously [58]. STAT1^-/-^ mice were backcrossed onto the 129SVEV background as previously published [63]. Proper genetic background of STAT1^-/-^ mice was additionally assessed at the DartMouse Speed Congenic Core Facility as previously published [64]. 129SVEV (129S6/SvEvTac) mice were purchased from Taconic labs and bred in house. Mice expressing TdTomato following Cre-mediated recombination (Ai14 mice; B6.Cg-Ct(ROSA)26Sor^tm14(CAG-tdTomato)Hze^/J) were purchased from Jackson Laboratories and generously provided by Hermes Yeh (Geisel School of Medicine). Stat1-floxed (Stat1^fl/fl^) mice were generously provided by Floyd Wormley (UT San Antonio), and generated by Mathias Müller [65]. Nestin-cre (B6.Cg-Tg(Nes-cre)1Kln/J) mice were purchased from Jackson Laboratories and described previously [66]. Nestin-cre mice were maintained as a hemizygous line. Progeny from Nestin-cre Stat1-flox crosses were genotyped prior to use.

### Antibodies and reagents

Primary antibodies used were rabbit anti-HSV-1 (B0114, Dako), chicken anti-NeuN (ab134014, Abcam), mouse anti-NeuN (clone A60, Millipore), and rabbit anti-beta III Tubulin (ab18207, Abcam), rabbit anti-Iba1 (Wako), rabbit anti-STAT1α91 (M-23, Santa Cruz Biotechnology). The mouse anti-A5 antibody (FE-A5), developed by Bruce A. Fenderson at Thomas Jefferson University, was obtained from the Developmental Studies Hybridoma Bank, created by the NICHD of the NIH and maintained at The University of Iowa, Department of Biology, Iowa City, IA. Secondary antibodies used were goat-anti-mouse/rabbit Alexa 555, goat-anti-mouse/rabbit Alexa 488 (Invitrogen), and donkey-anti-chicken Alexa488 (Jackson ImmunoResearch Laboratories). Isolectin B4 conjugated to FITC (Sigma) was added to cultures at a concentration of 10μg/mL to stain KH10 neurons in chambers [67]. Counterstaining was done by incubation with DAPI (Invitrogen). Samples were mounted using FluorSave Reagent (Calbiochem). When indicated, cells were treated with 12.5U/mL (unless noted) IFNβ (PBL Interferon Source), 100ng/mL IFNγ (Miltenyi Biotec) or 100ng/mL IFNλ2 (PeproTech) for 18 hours prior to infection or for the specified amount of time prior to staining.

### HSV genomic copy number quantification

Neurons grown in chambers were pretreated with 100μM Acyclovir (ACV, Spectrum) for 18 hours, and infected axonally in the presence of ACV. The DNA from the soma compartment was harvest at indicated times post infection in TRIzol (Ambion) per manufacturer’s instructions through use of back extraction buffer (4M guanidine thiocyanate, 50mM sodium citrate, 1M Tris). Copy number was determined through qPCR for the viral thymidine kinase (tk) normalized to the single-copy mouse adipsin as described previously [68]. A standard curve for tk was prepared using HSV bacterial artificial chromosome (BAC) 17–49 [69]. Mouse genomic material was used for adipsin standard curves. As a control, the genomic copy number of strain 17 versus Δγ34.5 viral stocks was empirically determined to be equivalent as judged by the above PCR protocol.

### RT-qPCR

Samples were harvested in TRIzol (Ambion) and RNA extracted per manufacturer instructions. RNA was treated with DNA-free kit (Ambion) and cDNA synthesized using the SuperScriptIII Reverse transcriptase kit (Invitrogen) with random primers (Promgea). For qPCR, SYBR Select Master Mix (Life Technologies) was used with primers for IFIT1 (Fw: TGC TTT GCG AAG GCT CTG AAA GTG, Rv: TGG ATT TAA CCG GAC AGC CTT CCT), ISG15, (Fw: TGA GCA TCC TGG TGA GGA ACG AAA, Rv: AGC CAG AAC TGG TCT TCG TGA CTT) and 18s (Fw: TCA AGA ACG AAA GTC GGA GG, Rv: GGA CAT CTA AGG GCA TCA CA). IFIT1 and ISG15 values were calculated by the 2-ΔΔ^CT^ method [70] normalized to 18s, and values for IFN treated cells were normalized to mock.

### Immunofluorescence and histological analysis

Mice were perfused with PBS followed by 4% formaldehyde. Brain or trigeminal ganglia were embedded in OCT (Tissue-Tek) and 15μm sections taken on a Leica CM1860 cryostat. Tissue sections or fixed neuron cultures were incubated in 0.1% Triton-X100 (Sigma) in 5% normal goat serum (NGS- Vector Laboratories) in PBS for 1 hour. Primary and secondary antibody incubations were done in 2% NGS/0.1% Triton overnight at 4°C and for 1 hour at room temperature, respectively. Staining with A5 primary antibody was done at 4°C for 48 hours. Fixed cultures/tissue were imaged on Zeiss Axio Observer inverted microscope and montages created using motorized stage and ZEN software. Images were analyzed using ImageJ/Fiji with a minimum of 4 sections per TG and a minimum of 7 TGs per group. Quantification of neuronal subtype in compartmentalized chambers was done for a minimum of 3,000 neurons total per chamber over 2 experiments with 6 chambers total.

### Animal infection procedures

Mice were anesthetized intraperitoneally with ketamine (90 mg/kg) and xylazine (10 mg/kg). Corneas were bilaterally scarified and mice were inoculated by adding 2 × 10^6^ PFU per eye in a 5μl volume. Periocular disease was monitored over time as previously described [71]. For survival studies, mice were monitored over time and euthanized upon reaching endpoint criteria consisting of loss of more than 25% body weight and/or a drop in temperature by 3°C from baseline [72]. At indicated times following corneal infection, the following tissues were harvested and titers determined as previously described [58]; corneal swabs, periocular skin, trigeminal ganglia, brain, and brain stem. For periocular skin, two 6mm biopsy punches per eye were harvested and titers were averaged. All tissues were harvested and stored at −80°C until processing. Tissues were mechanically disrupted and sonicated, and titers were determined via standard plaque assay on Vero cells as described previously [58].

### Ethics statement

This study was carried out in strict accordance with the recommendations in the Guide for the Care and Use of Laboratory Animals of the National Institutes of Health. The protocol was approved by the Dartmouth IACUC Committee (06/05/12, Permit Number: leib.da.1). No surgery was performed, and all efforts were made to minimize suffering.

## Supporting Information

S1 Fig Characterization of modified Campenot chambers. A) Immunofluorescence staining of KH10 (Isolectin B4, IB4) and A5 subtype neurons (green). DiI (red) was added to the axonal compartment, labeling neurons that extend axons through the central barrier. Scale bar = 50μm. B) Quantification of the percent of neurons with axons extending through the barrier (DiI+) that are IB4 or A5 positive. Error bars represent SEM of ≥5 chambers over 2 experiments. C) Mean fluorescent intensity (MFI) of supernatants from the soma and axon compartments collected 72 hours post-addition of a fluorescein-conjugated dextran dye (MW = 10,000) added to the axon compartment. Neurons were cultured in modified chambers for 2 weeks prior to the addition of dye. Each data point represents one chamber. Dashed line represents background MFI. Significance was evaluated by Student’s t-test where **p<0.01.
(TIF)

S2 Fig VSV replication after axonal IFN treatment at higher IFNβ concentrations. Titers of VSV in the soma compartment 24 hours post infection via the soma of 129SVEV neuron cultures. Cultures were untreated or treated with 100 U/mL IFNβ in the axon compartment 18 hours prior to infection. Error bars represent SEM of 2 chambers each.
(TIF)

S3 Fig Higher concentrations of IFNβ and lower viral inoculum does not affect capsid trafficking. HSV genome copy number of WT (strain 17) or Δγ34.5 virus measured from the soma compartment of axonally infected 129SVEV neuron cultures at 3hpi. Cultures were treated with 100μM ACV, and with 100 U/mL IFNβ in the axon compartment for 18 hours prior to infection with 10^6^ PFU. Error bars represent SEM of 3 chambers each.
(TIF)

S4 Fig Model of axonal versus soma IFN signaling. A) Axonal IFNβ signaling, through STAT1, upregulates a response capable of restricting titers of HSV-1 entering the neurons at distal axon terminals. Replication of virus entering the cell at the soma, however, is unaffected. B) IFNβ signaling at the soma leads to upregulation of a classical antiviral response via STAT1 translocation to the nucleus and ISG transcription. This response can restrict titers of HSV-1 entering at distal axons and locally at the soma.
(TIF)

S5 Fig Viral tropism in the TG. Immunofluorescence of TG sections from st17 infected Stat1^N-/-^ mice (A) and Δγ34.5 Stat1^fl/fl^ mice (B) 5 days post infection with 2 x 10^6^ PFU/eye virus via the cornea. As depicted in Fig 5, tissue sections show immunostaining for HSV antigen (red), the neuronal marker NeuN (green), and nuclei (DAPI, blue). The white arrow indicates an infected SGC distinguishable by morphology and proximity to a NeuN+ neuronal cell body. Scale bar = 10μm.
(TIF)

S6 Fig Antiviral response of TG neurons to Type I, II and III IFN. A) Immunofluorescence staining of TG neuron cultures for STAT1 (red), the neuronal marker NeuN (green) and nuclei (DAPI, blue). Cells were untreated (unt) or treated with IFNβ (100U/mL), IFNγ (100ng/mL), or IFNλ (100ng/mL) for 1 hour. Scale bar = 20μm. B) Titers of VSV in IFN-treated neuron cultures at 24hpi. Cells were untreated or treated with IFNβ, IFNγ or IFNλ as in (A) for 18 hours prior to infection with VSV. Error bars represent SEM of a minimum of 3 samples over 2 experiments. One-way ANOVA was performed where *p<0.05, ***p<0.001 compared to untreated, and § p<0.001 between 129SVEV and STAT1^-/-^ within treatment groups.
(TIF)
