# Supplementary figures and images for: Neuronal Interferon Signaling Is Required for Protection against Herpes Simplex Virus Replication and Pathogenesis
Source: PLoS Pathog. 2015 Jul 8;11(7):e1005028. doi: 10.1371/journal.ppat.1005028 (PMC4495997; doi:10.1371/journal.ppat.1005028)

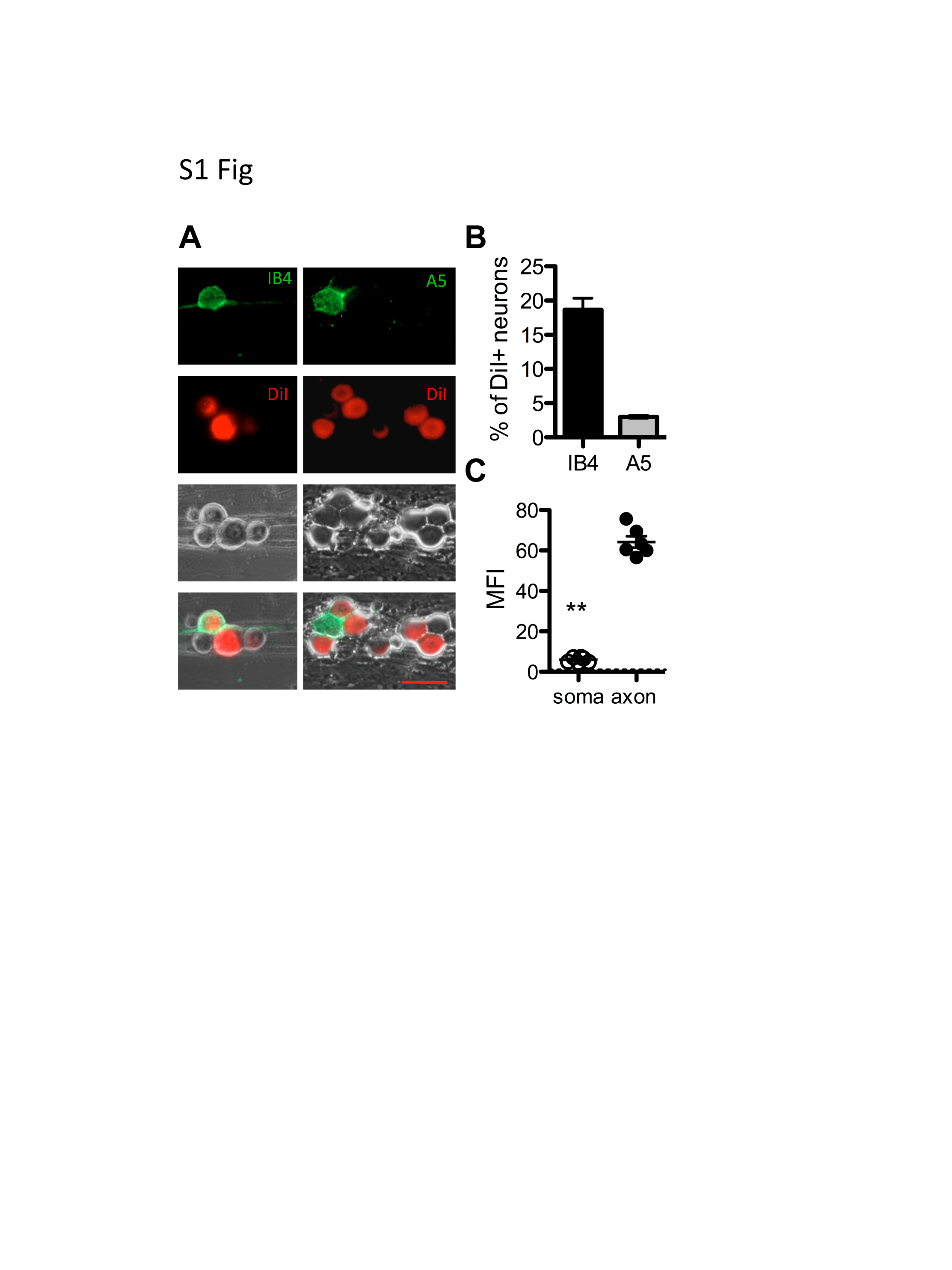

Supplement: S1 Fig — A) Immunofluorescence staining of KH10 (Isolectin B4, IB4) and A5 subtype neurons (green). DiI (red) was added to the axonal compartment, labeling neurons that extend axons through the central barrier. Scale bar = 50μm. B) Quantification of the percent of neurons with axons extending through the barrier (DiI+) that are IB4 or A5 positive. Error bars represent SEM of ≥5 chambers over 2 experiments. C) Mean fluorescent intensity (MFI) of supernatants from the soma and axon compartments collected 72 hours post-addition of a fluorescein-conjugated dextran dye (MW = 10,000) added to the axon compartment. Neurons were cultured in modified chambers for 2 weeks prior to the addition of dye. Each data point represents one chamber. Dashed line represents background MFI. Significance was evaluated by Student’s t-test where **p<0.01. (TIF) [file ppat.1005028.s001.tif]

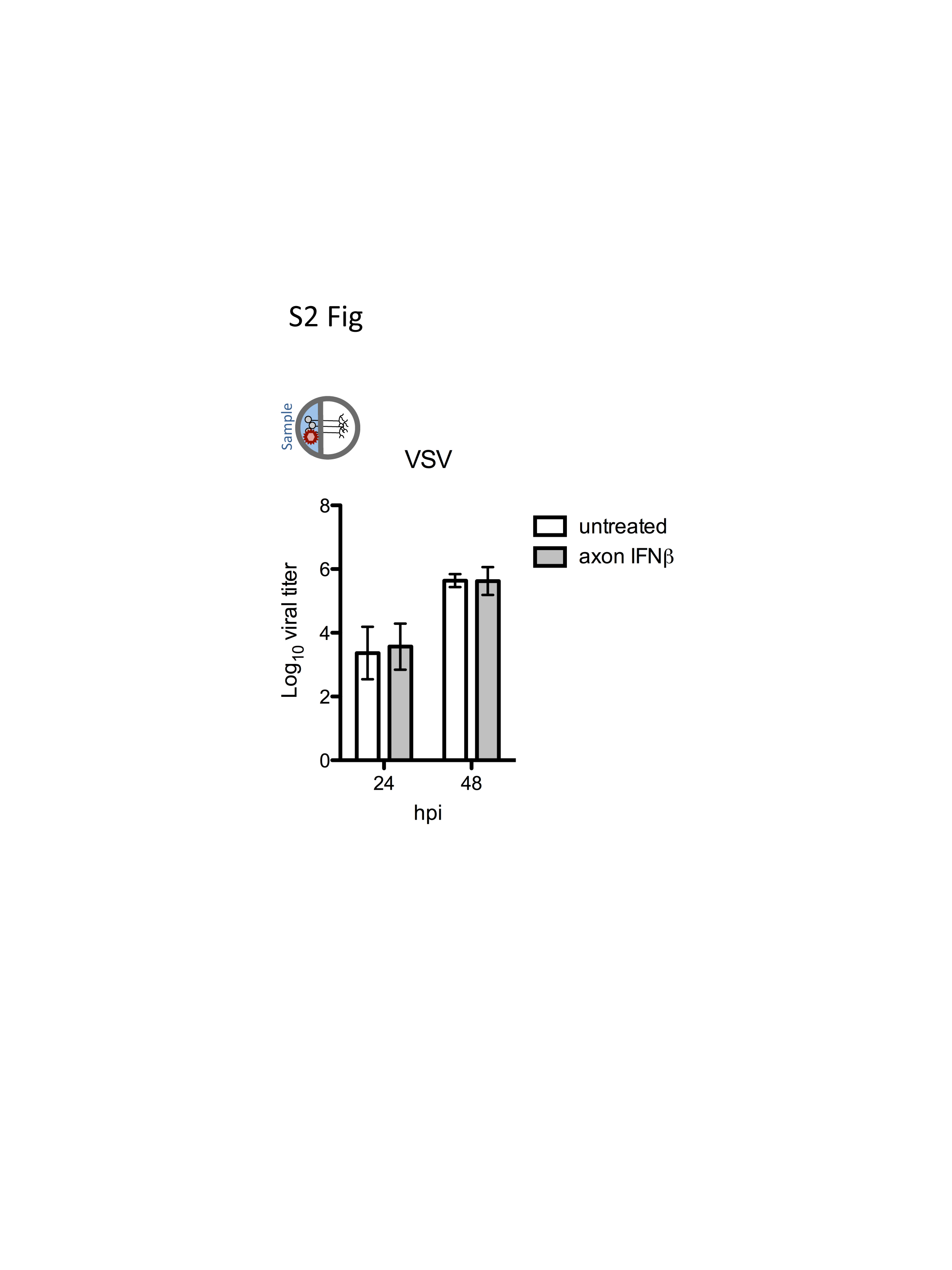

Supplement: S2 Fig — Titers of VSV in the soma compartment 24 hours post infection via the soma of 129SVEV neuron cultures. Cultures were untreated or treated with 100 U/mL IFNβ in the axon compartment 18 hours prior to infection. Error bars represent SEM of 2 chambers each. (TIF) [file ppat.1005028.s002.tif]

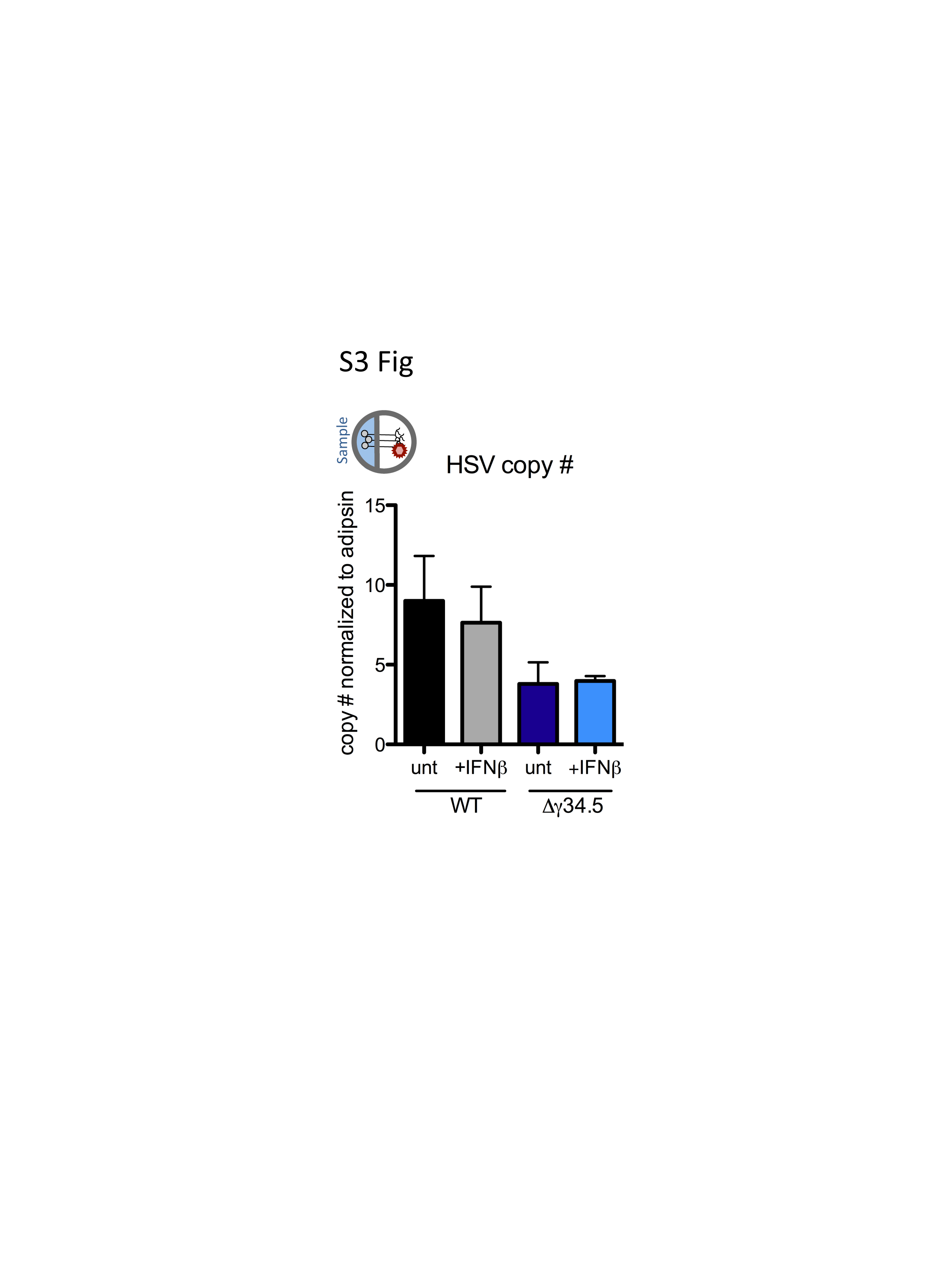

Supplement: S3 Fig — HSV genome copy number of WT (strain 17) or Δγ34.5 virus measured from the soma compartment of axonally infected 129SVEV neuron cultures at 3hpi. Cultures were treated with 100μM ACV, and with 100 U/mL IFNβ in the axon compartment for 18 hours prior to infection with 106 PFU. Error bars represent SEM of 3 chambers each. (TIF) [file ppat.1005028.s003.tif]

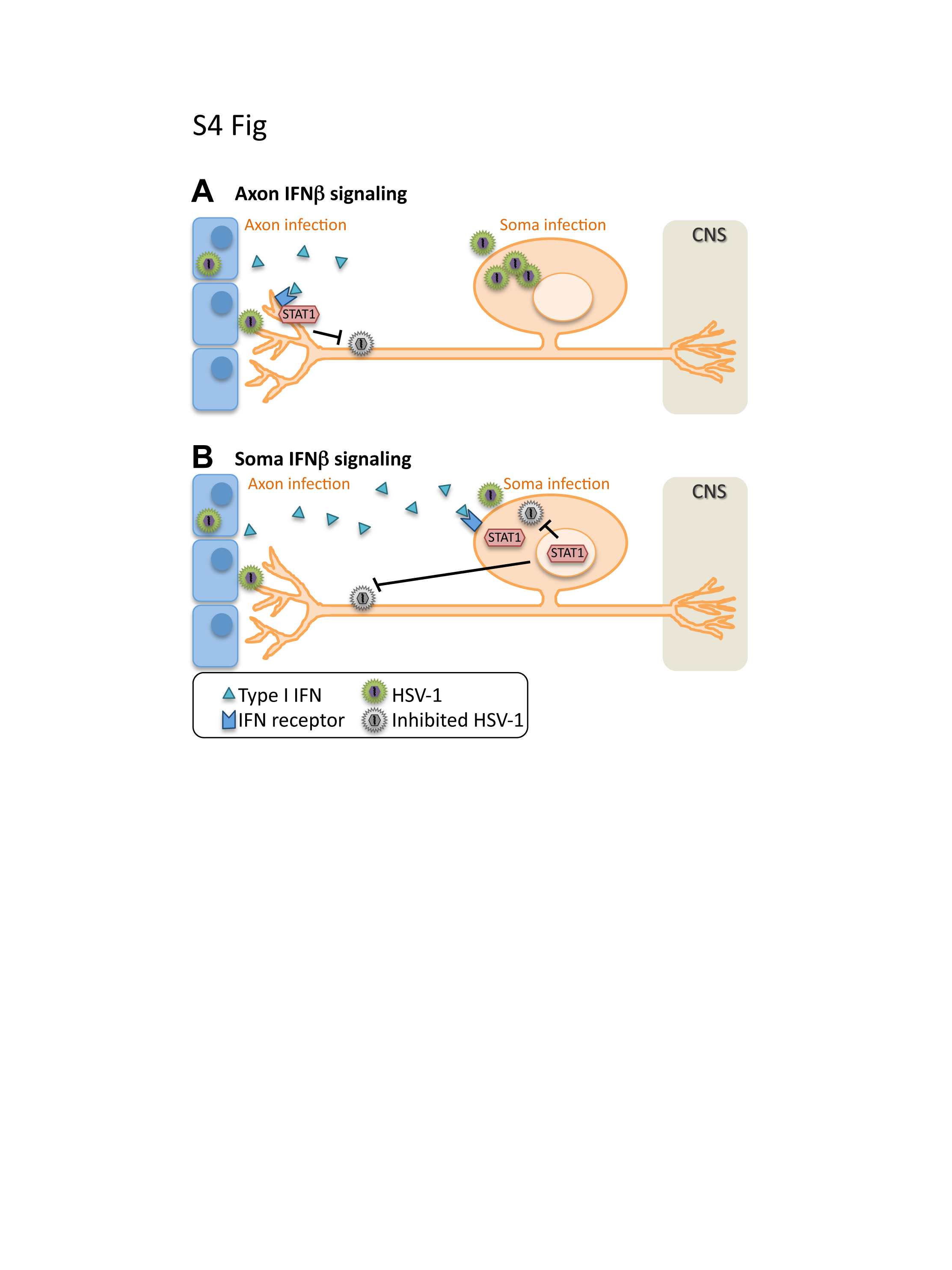

Supplement: S4 Fig — A) Axonal IFNβ signaling, through STAT1, upregulates a response capable of restricting titers of HSV-1 entering the neurons at distal axon terminals. Replication of virus entering the cell at the soma, however, is unaffected. B) IFNβ signaling at the soma leads to upregulation of a classical antiviral response via STAT1 translocation to the nucleus and ISG transcription. This response can restrict titers of HSV-1 entering at distal axons and locally at the soma. (TIF) [file ppat.1005028.s004.tif]

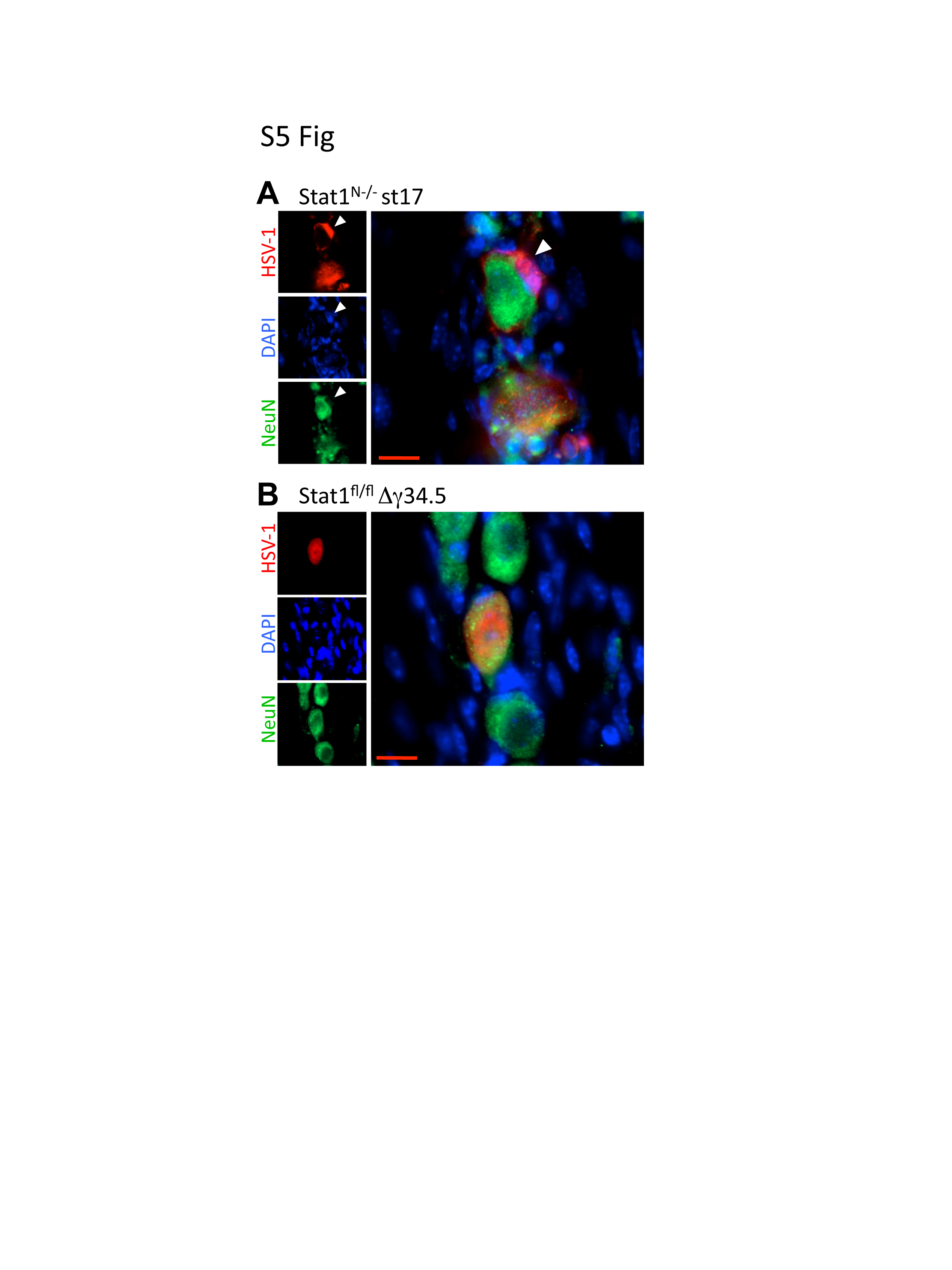

Supplement: S5 Fig — Immunofluorescence of TG sections from st17 infected Stat1N-/- mice (A) and Δγ34.5 Stat1fl/fl mice (B) 5 days post infection with 2 x 10^6^ PFU/eye virus via the cornea. As depicted in Fig 5, tissue sections show immunostaining for HSV antigen (red), the neuronal marker NeuN (green), and nuclei (DAPI, blue). The white arrow indicates an infected SGC distinguishable by morphology and proximity to a NeuN+ neuronal cell body. Scale bar = 10μm. (TIF) [file ppat.1005028.s005.tif]

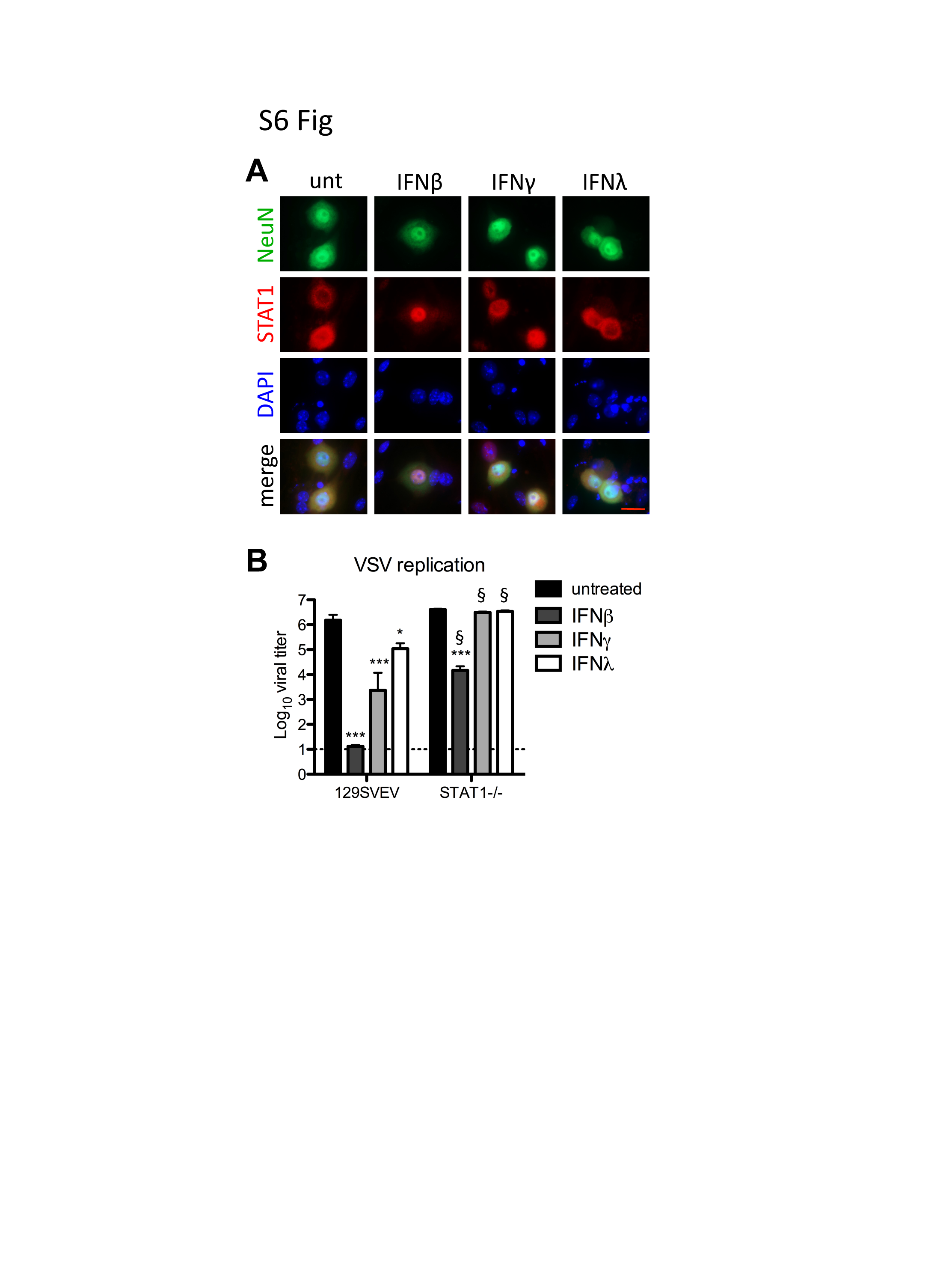

Supplement: S6 Fig — A) Immunofluorescence staining of TG neuron cultures for STAT1 (red), the neuronal marker NeuN (green) and nuclei (DAPI, blue). Cells were untreated (unt) or treated with IFNβ (100U/mL), IFNγ (100ng/mL), or IFNλ (100ng/mL) for 1 hour. Scale bar = 20μm. B) Titers of VSV in IFN-treated neuron cultures at 24hpi. Cells were untreated or treated with IFNβ, IFNγ or IFNλ as in (A) for 18 hours prior to infection with VSV. Error bars represent SEM of a minimum of 3 samples over 2 experiments. One-way ANOVA was performed where *p<0.05, ***p<0.001 compared to untreated, and § p<0.001 between 129SVEV and STAT1-/- within treatment groups. (TIF) [file ppat.1005028.s006.tif]
